# Research on the mechanism of plant root protection for soil slope stability

**DOI:** 10.1371/journal.pone.0293661

**Published:** 2023-11-27

**Authors:** Tingting Cao, Haiou Zhang, Tianqing Chen, Chenxi Yang, Jian Wang, Zhen Guo, Xubo Sun

**Affiliations:** 1 Shaanxi Provincial Land Engineering Construction Group Co. Ltd., Xi’an, China; 2 Institute of Land Engineering and Technology, Shaanxi Provincial Land Engineering Construction Group Co., Ltd., Xi’an, China; 3 Shaanxi Key Laboratory of Land Consolidation, Xi’an, China; 4 Key Laboratory of Degraded and Unused Land Consolidation Engineering, The Ministry of Natural Resources, Xi’an, China; University of Sharjah, UNITED ARAB EMIRATES

## Abstract

In order to investigate the impact of herbaceous root development on soil slope stability in expansive soil areas, the research was conducted in the soil slope experimental area of Yaoshi Town, Shangzhou District, Shangluo City. Three types of herbaceous plants, namely Lolium perenne, Medicago, and Cynodon dactylon, were planted to examine their influence on slope stability. The results indicated that Lolium perenne had significantly higher root length density and root surface area density compared to Cynodon dactylon and Medicago. However, the root weight density of Cynodon dactylon was found to be highest. The roots of Lolium perenne, Cynodon dactylon, and Medicago were predominantly observed in diameter ranges of 0 < L ≤ 1.0 mm, 0 < L ≤ 2.5 mm, and 2.5 < L ≤ 3.0 mm, respectively. The roots of herbaceous plants have the ability to enhance water retention in soil, resist hydraulic erosion of slope soil, and reduce soil shrinkage and swelling. During the initial phase of herbaceous planting, there is an accelerated process of organic carbon mineralization in the soil. The roots of herbaceous plants play a crucial role in soil consolidation and slope protection. They achieve this by dispersing large clastic particles, binding small particles together, altering soil porosity, enhancing soil water retention, and reducing soil water infiltration. It was found that Lolium perenne and Medicago, which have well-developed roots, exhibited superior slope protection effects. These findings contribute to the theoretical understanding for the implementation of green ecological protection technology on soil slopes.

## Introduction

Swelling soils are a type of soil that is highly unstable in terms of water absorption and swelling, water loss and shrinkage, attenuation of water bearing capacity and development of dry shrinkage fissures. With a high clay mineral content of swelling soil, the clay minerals of the expanded clay are mainly montmorillonite, illite, and kaolinite, most of which are Cenozoic sediments. It is a highly plastic clay. swelling and shrinking properties are due to the unique clay mineral composition and microstructural characteristics of swelling soil, making water-soil interaction, forming a macroscopic phenomenon in which the soil becomes swollen and soft after absorbing water, and shrinks after losing water, which is the main reason for the engineering problems of swelling soil [1]. Soils on slopes in swelling soil areas are often in an alternating state of dryness and wetness under natural precipitation and evaporation conditions. Soils swell when they are wet and shrink when they are dry, making the expansion and contraction process and its resulting hazards are more obvious [2–4]. Soils are very sensitive to changes in water content due to the presence of clay minerals such as montmorillonite and illite. Evaporation, rainfall and transient changes in water content and level can cause the soil to swell and shrink [5–7]. In the process of water absorption, the soil humidity increases, the volume expands and forms expansion pressure. In the process of dewatering, the soil will undergo axial and radial shrinkage, and its shrinkage strain gradually increases, which will eventually lead to cracks in the soil [8–10]. As the water content decreases, the area, length and density of the cracks show an increasing trend, which are significantly harmful to slope engineering and greening projects [11]. In slope engineering, when slope absorbs water, the soil swells, softens and loses strength. When the slope loses water the soil shrinks and consequently fissures are created. Repeated expansion and contraction lead to the loosening of the swelling soil and t form many irregular fissures in it, thus creating conditions for further erosion of the soil surface [12–15]. In the event of rainfall infiltration, it is very likely to lead to geological disasters such as landslides[16–20]. In the greening projects, cracks resulting from soil shrinkage may damage the root of herbaceous plants, thus affecting their distribution and water absorption and causing mechanical tearing [21]. On the other hand, soil shrinkage cracks will lead to changes water movement and solute migration characteristics in soil, and even generate preferential flow, causing ecological problems such as nutrient loss and groundwater pollution [22–24]. From this, it can be seen that the expansion and contraction of swelling soils is a hot issue in soil erosion research.

Our country has one of the largest areas of expansive soil in the world, covering approximately 100,000 km^2^. It is mainly distributed in the middle and lower Yellow River area, the middle and lower Yangtze River area, and some coastal areas in the southeast, including Guangdong, Sichuan, Shaanxi, and other provinces [25]. The expansive soil area in Shaanxi Province spans approximately 1300 km^2^, which makes up roughly 6% of the province’s total area. This type of soil is primarily found in the hilly region of southern Shaanxi and is heavily concentrated in areas with terraced fields [26]. The administrative regions that are most affected by expansive soil are Ankang, Shangluo, and Hanzhong. In this region, heavy and intense precipitation, combined with a thin soil layer and limited water capacity, results in reduced soil water absorption and expansion strength. This leads to the formation of cracks and subsequent collapse of terrace terraces, slope collapse, and significant soil and water loss. These factors also contribute to a sharp reduction in the cultivated land area, waste of available land resources, and pose a direct threat to regional agricultural production and farmers’ income.

Several studies have demonstrated that the twisting and strengthening of plant roots in soil can impede the expansion and contraction of the soil [27–32]. As a solution for slope protection, green ecological models have been utilized in engineering applications. The special report by Intergovernmental Panel on Climate Change (IPCC, 2019) has urged policy makers to increase vegetation plantation in order to combat global CO_2_ levels [33]. Plant slope protection engineering is an effective and aesthetically pleasing method for safeguarding slopes while also benefiting the environment and economy [34–37]. Studies have demonstrated that the survival of plants can influence soil water retention and alter the flow of water within soil [38, 39]. The impact and mechanism of plant growth on slope protection in areas with expansive soil, where slope disasters are common, have not been extensively studied and quantified [40–43]. To address this, our research group selected the low hilly region of southern Shaanxi, where expansive soil is prevalent, as our research area. We established experimental fields in the Shangluo area of Shaanxi Province to investigate this issue. The study focused on the protection mechanism of slope soil in expansive soil areas. Three types of herbs, namely Medicago, Lolium perenne, and Cynodon dactylon, which are suitable for growth on soil slopes, were selected to investigate the mechanism of plant root protection on soil slope stability in expansive soil areas. The main objectives of this study are as follows: 1) To examine the root characteristics of different herbaceous plants on soil slopes in expansive soil areas. 2) To explore the effects of different root stages on slope soil properties such as water-holding capacity, swelling and shrinkage, soil nutrients, soil texture, and soil porosity in expansive soil areas. 3) To investigate the effects of different plant roots on soil microstructure in expansive soil areas. This study investigates the obstruction mechanism of various herb roots on soil expansion and contraction in expansive soil areas from both macro and micro perspectives. It reveals the mechanism of soil consolidation and slope protection by different herb roots in expansive soil areas. Additionally, it identifies plant species that are beneficial for soil conservation and slope protection in this region. These findings provide a scientific theoretical basis for the application of green ecological slope protection technologies in similar areas.

## Materials and methods

### Overview of the experimental area

**Fig 1:**
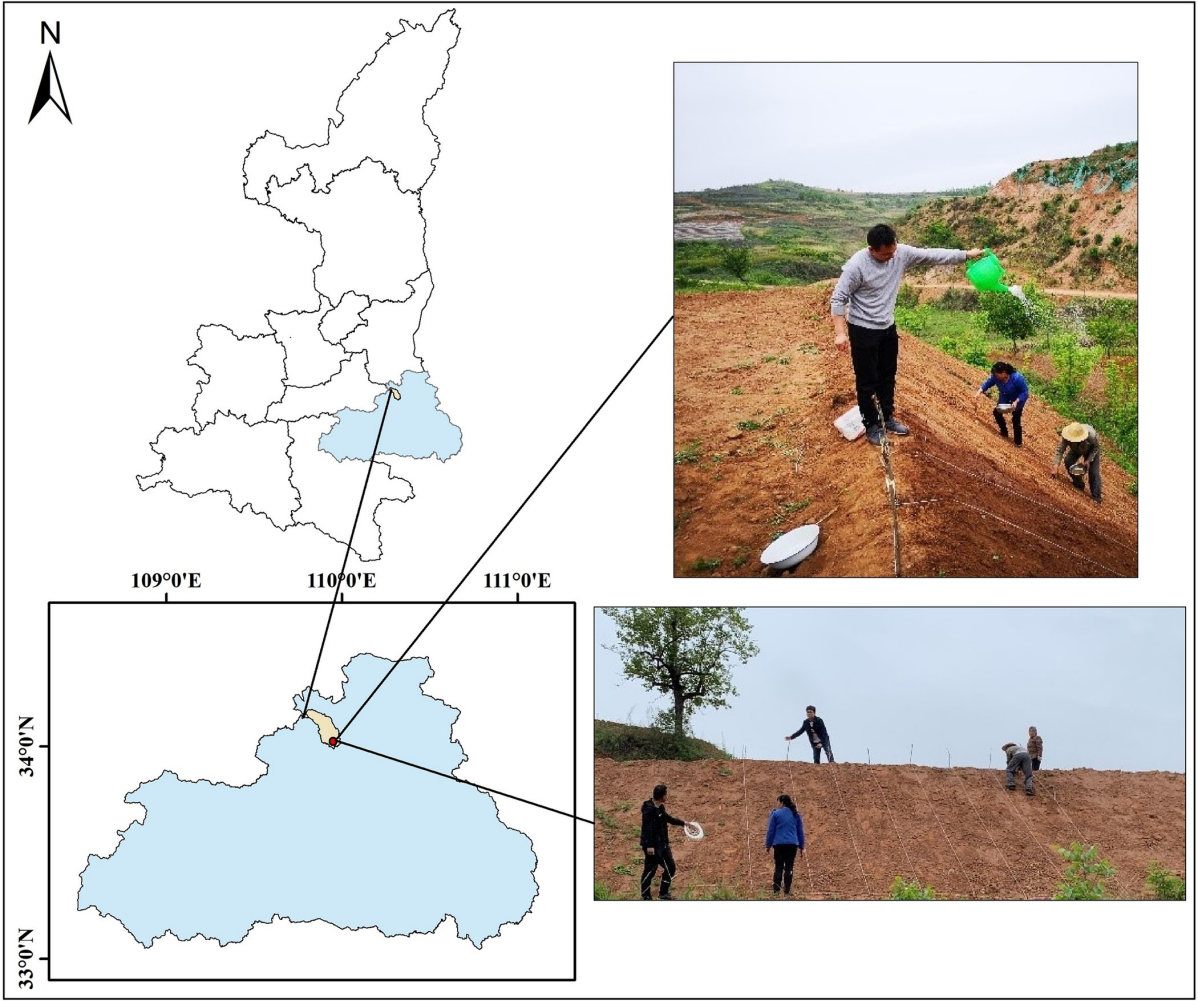
Research area location map. (Yaoshi Town, Shangluo City, Shaanxi Province, China).

**Fig 2:**
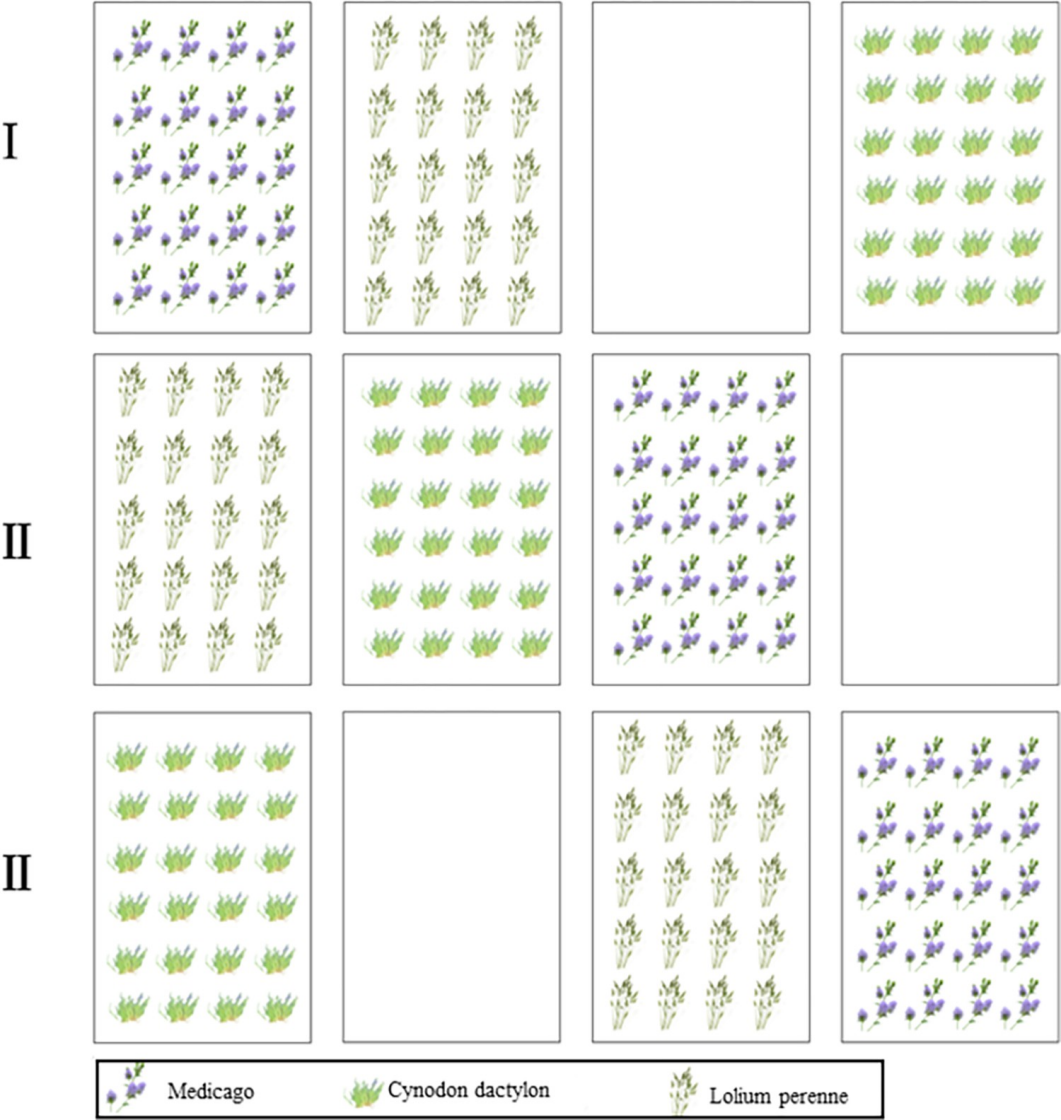
The distribution map for the division of the test area.

The study was conducted in Shangzhou District, Shangluo City, Shaanxi Province (Fig 1), an area with a temperate monsoon climate and complex terrain, including deep mountain gullies, ravines, valleys, overlapping mountains, and barriers, resulting in significant differences in vertical height. A field plot experiment was conducted with a consistent slope of 38° and a height of 3m for different treatments. The experimental field is under the Key Laboratory of Degraded and Unused Land Improvement Engineering, Ministry of Natural Resources, and all researchers involved in the study are part of the laboratory. The trial was initiated in April 2021.Three herbaceous plants, Medicago, Cynodon dactylon and Lolium perenne, were selected and planted in subzones, and a control area was set up. Each plot was set at 6 m x 4 m. Three replications were set up in each trial area. Medicago, Cynodon dactylon and Lolium perenne planted at a spacing of 30 cm x 35 cm. The distribution map for the division of the test area is illustrated in Fig 2. During the cultivation period, regular weeding and watering were carried out in each zone. After a year and a half of growing cycles, the team sampled the plants in September 2022.

### Sample collection and testing

Sampling was carried out in September when the plant cover had reached 80% after planting and cultivation. Sampling points were set up in each sample plot according to an S-shaped curve, with the above-ground parts of the plants removed. the centre of the cutting ring coinciding with the centre, and the original soil samples were collected vertically downwards along the plants. The original soil sample was collected vertically downwards along the plant, with the plant as the central point and the centre of the cutting ring coinciding with the central point.

The original soil samples were sealed in plastic bags and brought back to the laboratory. The cutting ring samples were used to test the soil moisture characteristic curve, soil line shrinkage and soil swelling rate, and the original soil samples were used for scanning electron microscopy observation of microstructure.

The characteristic curve of soil moisture was determined using the soil centrifuge method, utilizing the Hitachi high speed cooling centrifuge (CR21) manufactured in Japan. A soil sample with fresh plant roots was placed into a centrifuge with a suction range of 0–10000 cm water column. The experiment used suctions of 10, 50, 100, 300, 500, 700, 1000, 3000, 5000, 7000, and 10000 cm water column, with corresponding equilibrium times of 10, 17, 26, 42, 49, 53, 58, 73, 81, and 85 minutes, respectively. The moisture content of soil was determined by weighing the sample after each centrifugation, and then converted to volume moisture content. The shrinkage amount of the soil sample in the cutting ring was measured using a vernier caliper. During the dewatering process, the soil inside the cutting ring undergoes axial shrinkage, which results in a decrease in the sample height. In this research, the shrinkage characteristics of soil were evaluated by selecting only the axial shrinkage index, as axial shrinkage was the only type of shrinkage observed in the soil inside the cutting ring during the experiment. The extent of vertical deformation of the soil can be measured by the dimensionless geometric factor δ [44]. The evaluation of soil axial shrinkage characteristics can be determined using the following formula:

$$\delta_{s}=(h_{0}-h)/h_{0}\times 100\%$$ (1)

In the formula: *δ*_*s*_*—*axial shrinkage strain, %; *h*_*0*_—initial height of soil mass, mm;

The WinRHIZ0 system, developed in Canada, was used to measure the root properties of the plant. An Epson Perfection V800 photo scanning device was also employed in this process. To obtain the plant roots, soil samples in the cutting ring were cleaned with water multiple times. The resulting roots were then placed in a root tray with an appropriate amount of water, expanded with tweezers, and scanned to obtain images. The root characteristics including length, surface area, diameter levels, number of tips, forks, crossings and other parameters were analyzed using the WinRHIZO root analysis system. The roots were then dried in paper envelopes at 105°C for 72 hours to obtain the root biomass. Root length density, root surface area density,root weight density is calculated as follows [45]:

Rootlengthdensity=lengthofrootsystemincuttingring/cuttingringvolume (2)

Rootsurfaceareadensity=rootsurfaceareaincuttingring/cuttingringvolume (3)

Rootweightdensity=driedrootbiomassincuttingring/cuttingringvolume (4)

The expansion rate of soil was determined using the non-load expansion rate test method outlined in the GB/T 50123–2019 geotechnical test method standard. The test was conducted using a dilatometer. The soil sample was tested using a ring knife with a sample thickness of 10 mm. After installing the instrument, distilled water was added to the water box and the soil was allowed to absorb and expand through capillary suction. The first 15 minutes were recorded with a thousandmeter reading taken every 0.5 minutes. Subsequently, readings were taken every 1 minute until the thousandmeter reading remained constant. This indicated that the soil had reached its maximum expansion. The instantaneous and final expansion rates of the soil were then calculated using Eqs (5) and (6).


$$\delta_{p}=(H_{t}-H_{0})/H_{0}\times 100\%$$ (5)

$$\delta_{f}=(H_{f}-H_{0})/H_{0}\times 100\%$$ (6)

In the formula: *H*_*t*_*—*the expansion height of soil at a given time, mm; *H*_*0*_—initial height of soil mass, mm; *H*_*f*_*—*final saturation height of soil, mm.

The soil samples underwent a process of drying, gold spraying, and coating before being studied under scanning electron microscopy (SEM) to observe and analyze their micromorphology [46]. The capillary porosity, non-capillary porosity and total porosity were measured by the cutting ring method. Organic matter was determined by the potassium dichromate-concentrated sulfuric acid external heating method, and soil texture was determined by the densitometer method.

### Statistical analyses

The statistical significance of different plant root characteristics and soil swelling and shrinkage characteristics was assessed using Fisher’s least significant difference (LSD). The relationship between root characteristics of different plants and soil expansion and contraction was analyzed by spearman. All statistical analyses were performed using R.3.3.2 and Microsoft Excel 2019.

## Results

### Study of the root system characteristics of different plants

#### Root length density, root surface area density and root weight density of different plants

During the experiment, the root systems of different types of herbaceous plants in the test area were collected intact. The main root of Medicago was long, without branches, with few capillary fibrous roots. The main root was bent due to slope planting, and the root structure was relatively simple. The stolons of Cynodon dactylon are very long, and their root is strong and angled, with a root size between that of Medicago and Lolium perenne. The Lolium perenne is a clumping plant with a very well-developed and mainly superficial root system, numerous tillers and a slender and redundant root system that is difficult to separate from each other. Root length, root surface area and root weight indicators were tested by rinsing the soil inside the ring knife and isolating the root.

As can be seen from Table 1, after one year of plant cultivation, the root length density of Lolium perenne was significantly higher than that of Cynodon dactylon and Medicago, with Lolium perenne having 5.80 times the root length density of Cynodon dactylon and 9.88 times that of Medicago. The root surface area density of Lolium perenne was 3.07 times that of Cynodon dactylon and 4.45 times that of Medicago. The root weight density of Cynodon dactylon was 18.87% higher than that of Lolium perenne and 57.50% higher than that of Medicago.

**Table 1:** Root characteristics of different types of herbaceous plants.

Species	Root length density (cm/cm^3^)	Root surface area density (cm^2^/cm^3^)	Root weight density (mg/cm^3^)
Cynodon dactylon	4.51±0.12b	0.29±0.01b	0.63±0.03a
Lolium perenne	26.18±1.22a	0.89±0.05a	0.53±0.02b
Medicago	2.65±0.09c	0.20±0.02b	0.40±0.04c

**Fig 3:**
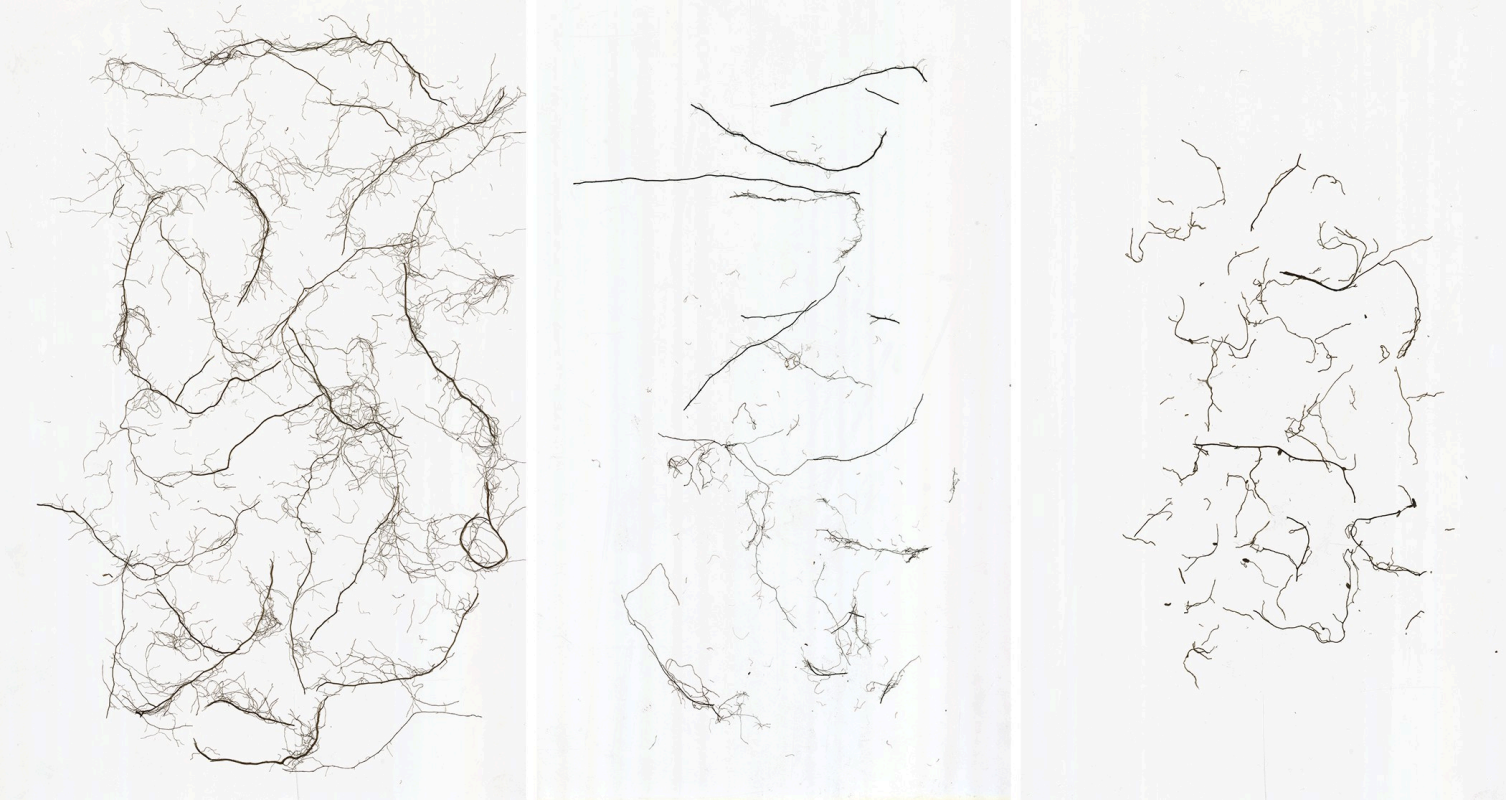
Scan of Lolium perenne(a), Cynodon dactylon(b) and Medicago(c).

Based on the analysis of the root morphology of the three plants, the significant differences in root length density, root surface area density and root weight density were due to the different root morphology of the three plants (Fig 3). The Lolium perenne has a well-developed root system with significantly higher root length and root surface area than the other plants, but its root is redundant and slender, and its root weight is slightly less than that of the Cynodon dactylon. The root length and root surface area of Cynodon dactylon are smaller than those of Lolium perenne due to its special root morphology, and therefore it is less able to bind to the soil than Lolium perenne. In comparison, Medicago has no significant advantage in terms of root length, root surface area and root weight.

#### Root diameter characteristics of different plants

The plant diameter classes were divided into six intervals: 0 < L ≤ 0.5, 0.5 < L ≤ 1.0, 1.0 < L ≤ 1.5, 1.5 < L ≤ 2.0, 2.0 < L ≤ 2.5 and 2.5 < L ≤ 3.0. By comparing the root diameter classes of the three herbaceous plants, the results are shown in Table 2. The root length of Lolium perenne was significantly higher than that of Cynodon dactylon and Medicago in the diameter class of 0 < L ≤ 0.5 mm. The root length of Cynodon dactylon was significantly higher than that of Lolium perenne and Medicago in the 0.5 < L ≤ 1.0 mm diameter range. In the 1.0 < L ≤ 2.5 mm diameter class range, Cynodon dactylon root length was significantly higher than the other plants, with Lolium perenne and Medicago having almost no root system in this diameter class range. Medicago had the longest root length in the 2.5 < L ≤ 3.0 mm diameter class range, with Cynodon dactylon and Lolium perenne having rarely root system in this diameter range.

**Table 2:** Distribution of root length at different diameter classes for three herbaceous.

Species	Root length at different root meridian levels(mm)					
	0<.L.< = 0.5	0.5<.L.< = 1.0	1.0<.L.< = 1.5	1.5<.L.< = 2.0	2.0<.L.< = 2.5	2.5<.L.< = 3.0
Cynodon dactylon	403.38±21.11b	36.29±2.08a	10.61±1.50a	0.52±0.08a	0.17±0.02a	0.02±0.01b
Lolium perenne	2607.14±240.21a	10.16±1.32b	0.01±0.00b	0.00	0.00	0.00
Medicago	257.41±23.24c	4.34±0.83c	0.06±0.00b	0.00	0.00	3.10±0.28a

#### The number of root tips, root forks and root crosses of different plants

As can be seen from the Table 3, Lolium perenne had the highest number of root tips, root forks and root crossings. The number of root tips of Lolium perenne was up to 9395, which was 2.54 times higher than that of Cynodon dactylon and 13.87 times higher than that of Medicago. The number of root forks of Lolium perenne was up to 18,569, which was 5.26 times higher than that of Cynodon dactylon and 19.52 times higher than that of Medicago. The number of root crossings of Lolium perenne was as high as 7452, which was 10.3 times that of Cynodon dactylon and 46.29 times that of Medicago.

**Table 3:** The number of root tips, root forks and root crosses of different plants.

Plant species	Numberof root tips	Number of root forks	Number of root crossings
Cynodon dactylon	3698±98b	3527±133b	723±60b
Lolium perenne	9395±164a	18569±494a	7452±363a
Medicago	677±30c	951±28c	161±23c

The total root length of the plant reflects the growth and elongation ability of the root system, the higher the number of root forks, the wider the distribution of the root system in the soil. The root tip reflects the physiological metabolic ability of the root system, the number of root crossings contains all the main and lateral root systems of the plant, the higher the number of root crossings, the larger the horizontal and vertical distribution area of the plant roots in the soil, which is more conducive to the root metabolism, development and growth, and the maximum soil fixation water retention capacity. In this study, the root scan showed that Lolium perenne had significantly more root length density, root surface area density, number of root tips, number of root forks, and number of root crossings than Medicago and Cynodon dactylon (P≤0.05).

### Study on the influence of different plant roots on soil slope stability

#### Study of soil water loss curves under the influence of different plant root

**Fig 4:**
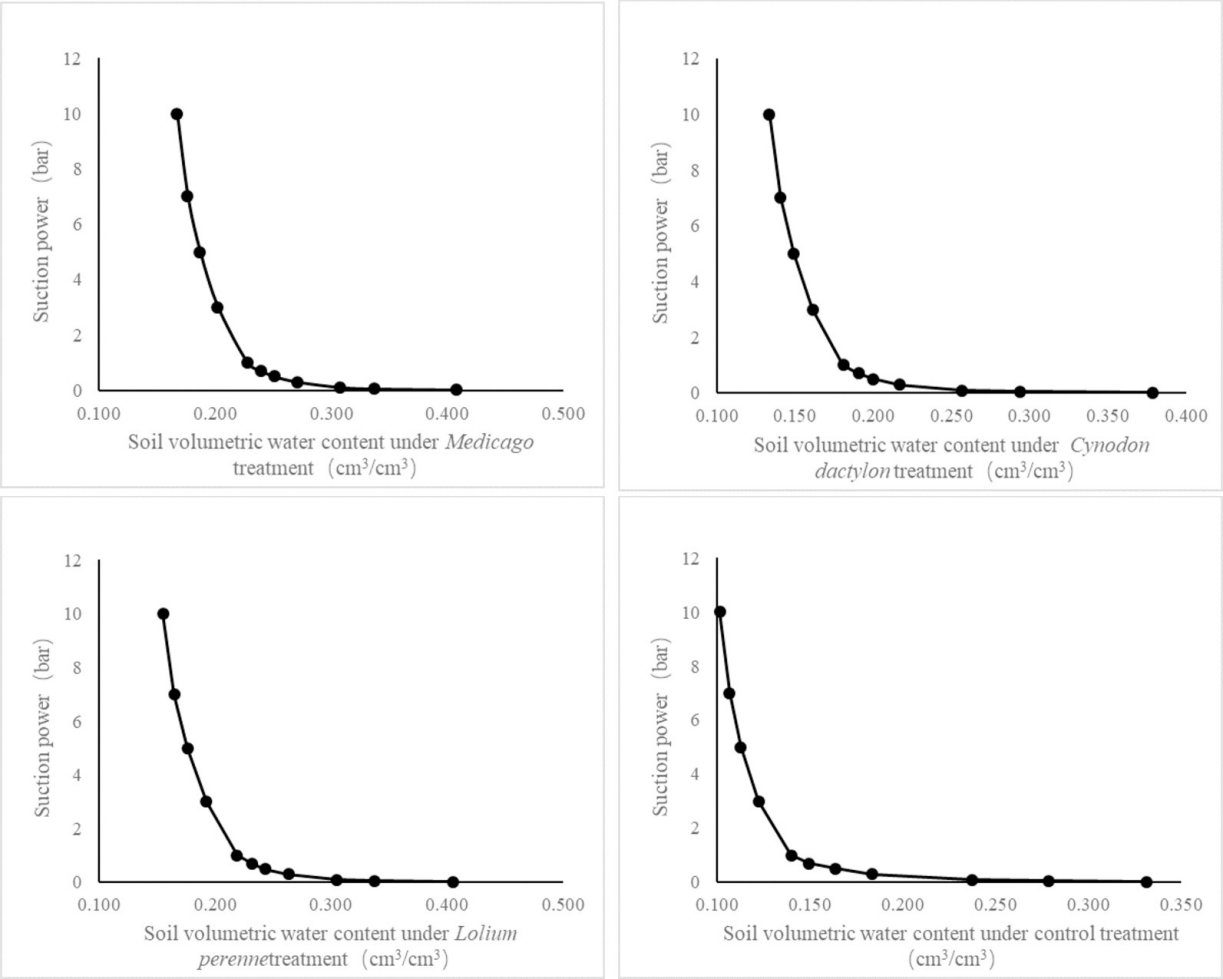
Soil moisture characteristics curve under different plant treatments. (a is for Medicago; b is for Cynodon dactylon; c is for Lolium perenne; d is for Control treatment).

By analysing the soil moisture characteristics curves under the four treatments (Fig 4), it can be seen that the soil moisture characteristics curves under the three treatments of Medicago, Cynodon dactylon and Lolium perenne were all on the right side compared to the control, and the range of deviation from the control varied according to the herbaceous plants.

The water-holding capacity of the soil was much greater with herbaceous plants than with the control treatment. The soil water-holding capacity of the different treatments showed the following trend: Medicago > Lolium perenne > Cynodon dactylon > control. At a minimum suction force of 0.01 bar, the soil volumetric water content increased by 22.9% compared to the control in the Medicago treatment, 22.0% compared to the control in the Lolium perenne treatment and 14.2% compared to the control in the Cynodon dactylon treatment. At a maximum suction force of 10 bar, the soil volume water content increased by 63.7% compared to the control in the Medicago treatment, 52.0% compared to the control in the Lolium perenne treatment and 31.4% compared to the control in the Cynodon dactylon treatment.

#### Study of soil shrinkage characteristics under the influence of different plant roots

In order to investigate the effect of different plant roots on the amount of soil shrinkage, the amount of shrinkage was studied when soil shrinkage occurs under different suction values for different plant treatments. It was found that under several treatments, the soil shrank axially only and did not shrink radially under different suction forces.

**Fig 5:**
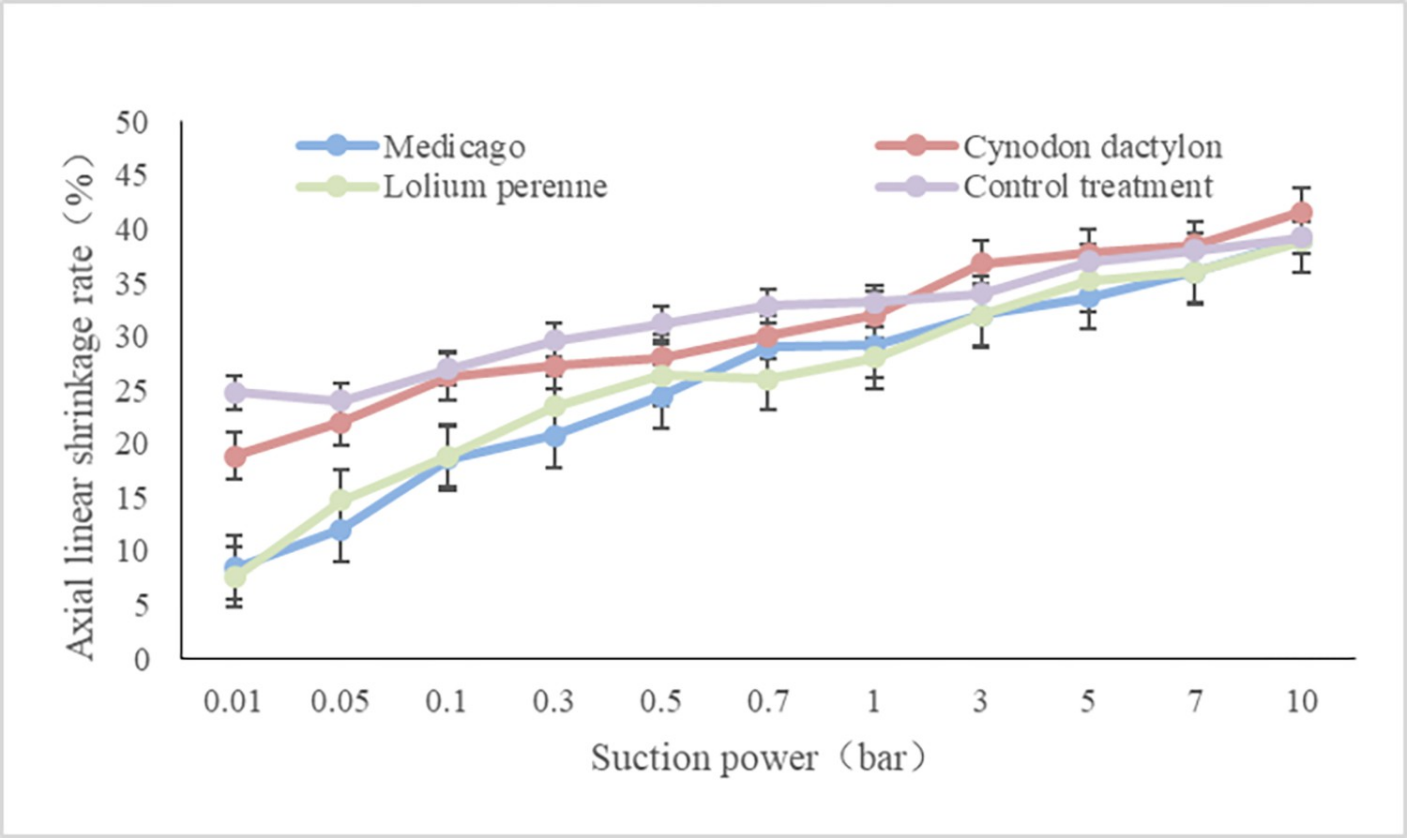
Axial line shrinkage of soil under different plant treatments.

As shown in Fig 5, after the test soil was saturated with water, the trend of axial linear shrinkage at 0.01 bar was control > Cynodon dactylon > Medicago > Lolium perenne in descending order. At a suction force of 0.05–0.5 bar, the trend of axial linear shrinkage for the different treatments was control > Cynodon dactylon > Lolium perenne > Medicago in descending order. At 3–10 bar, the trend of axial line shrinkage rate was Cynodon dactylon > control in descending order. There was no significant difference in line shrinkage between Medicago and Lolium perenne, which were both smaller than the control treatment.

It can be seen that there is a clear trend in soil shrinkage characteristics under different suction conditions under the influence of different plant roots. In the saturated state of the soil, after a slight application of suction, the linear shrinkage rate under all three plant treatments was less than that of the control treatment. The plant root system is a hindrance to the shrinkage properties of the soil.

#### Study of soil swelling characteristics under the influence of different plant roots

**Fig 6:**
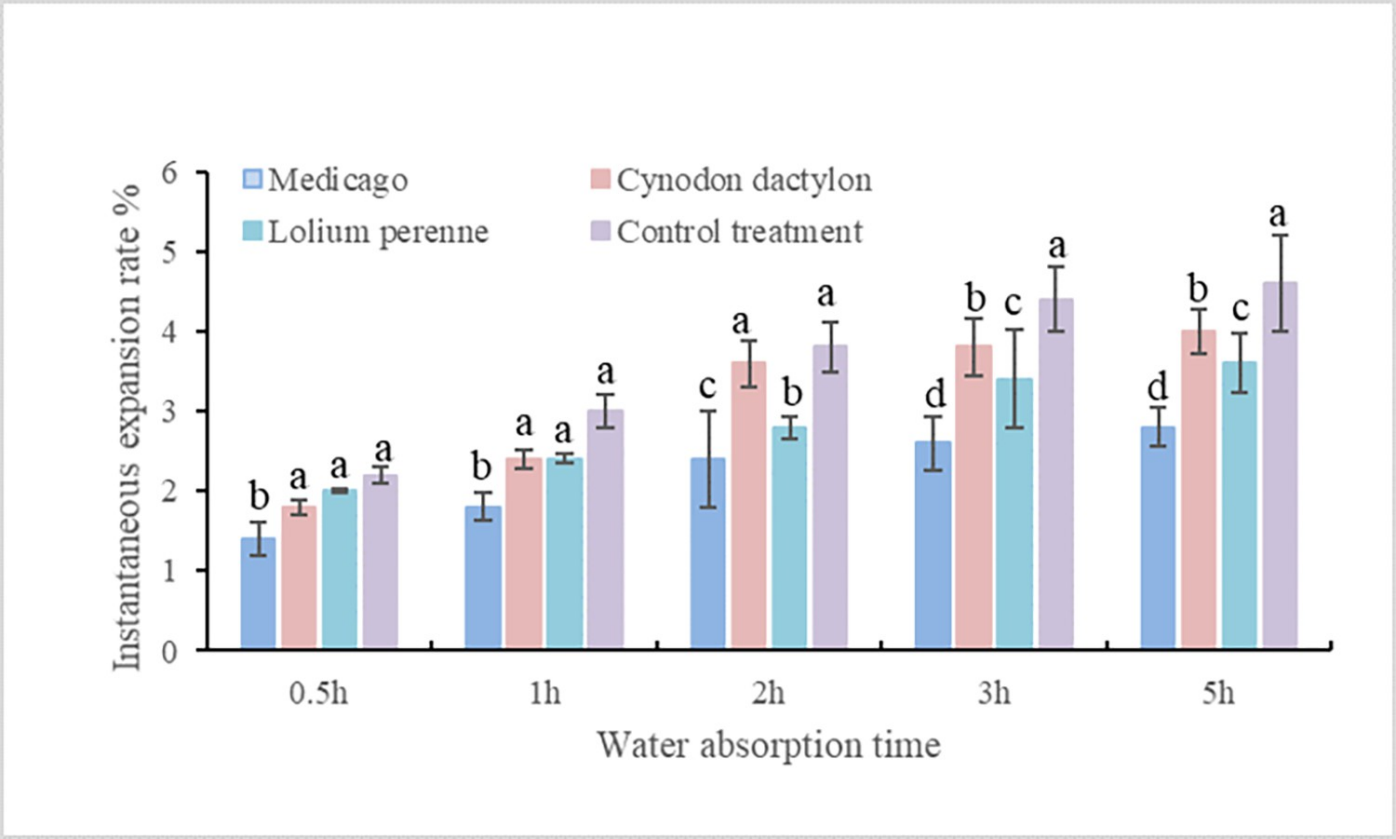
Transient swelling rate of soil under different plant treatments.

In order to analyze the swelling characteristics of the soil under different plant treatments, the instantaneous swelling rate was measured at different time periods. It was found that the soil under each treatment was basically close to saturation after 5h and no longer swelled. The results are shown in Fig 6, where the instantaneous swelling rate under the control treatment was higher than the three plant treatments at all time periods. The expansion rate was found to be reduced by 1.8% under Medicago, 0.6% under Cynodon dactylon, and 1.0% under Lolium perenne when compared to the control treatments.

#### Correlation analysis between plant roots and soil swelling and shrinking characteristics

**Fig 7:**
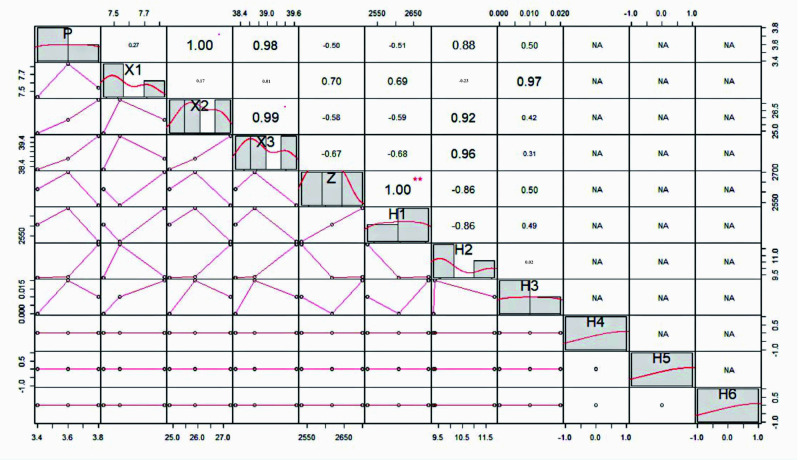
Correlation analysis between root diameter class and soil linear shrinkage and swelling rates under Lolium perenne treatment. P: swelling rate of soil; X1, X2, X3: the line shrinkage of the minimum, maximum and inflection points of suction; H1, H2, H3, H4, H5, H6: respectively denote the root lengths of 0<L< = 0.5mm, 0.5<L< = 1.0mm, 1.0<L< = 1.5mm, 1.5<L< = 2.0mm, 2.0<.L.< = 2.5mm, 2.5<.L.< = 3.0mm; Z:and total root systems; * p < 0.05 and ** p < 0.01.

In order to further clarify the influence of different plant root characteristics on soil swelling and shrinkage characteristics, the correlation between soil swelling and shrinkage characteristics and different diameter classes was analyzed under different plant treatments. In order to investigate the relationship between plant root warp classes and soil linear shrinkage characteristics, the minimum, maximum and inflection points of the line shrinkage under suction were deliberately screened for analysis. The results are shown in Fig 7.

As can be seen in Fig 7, the main diameter class range of Lolium perenne roots is 0 < L ≤ 0.5 mm, with a few roots at 0.5 < L ≤ 1 mm. There is no significant correlation between the root lengths of different diameter classes of Lolium perenne and the soil swelling rate. There is also no significant correlation between the root lengths of different diameter classes and the linear shrinkage rate of the soil under suction at the minimum, maximum and inflection points. In summary, Lolium perenne root size class does not affect its swelling and line shrinkage rates.

**Fig 8:**
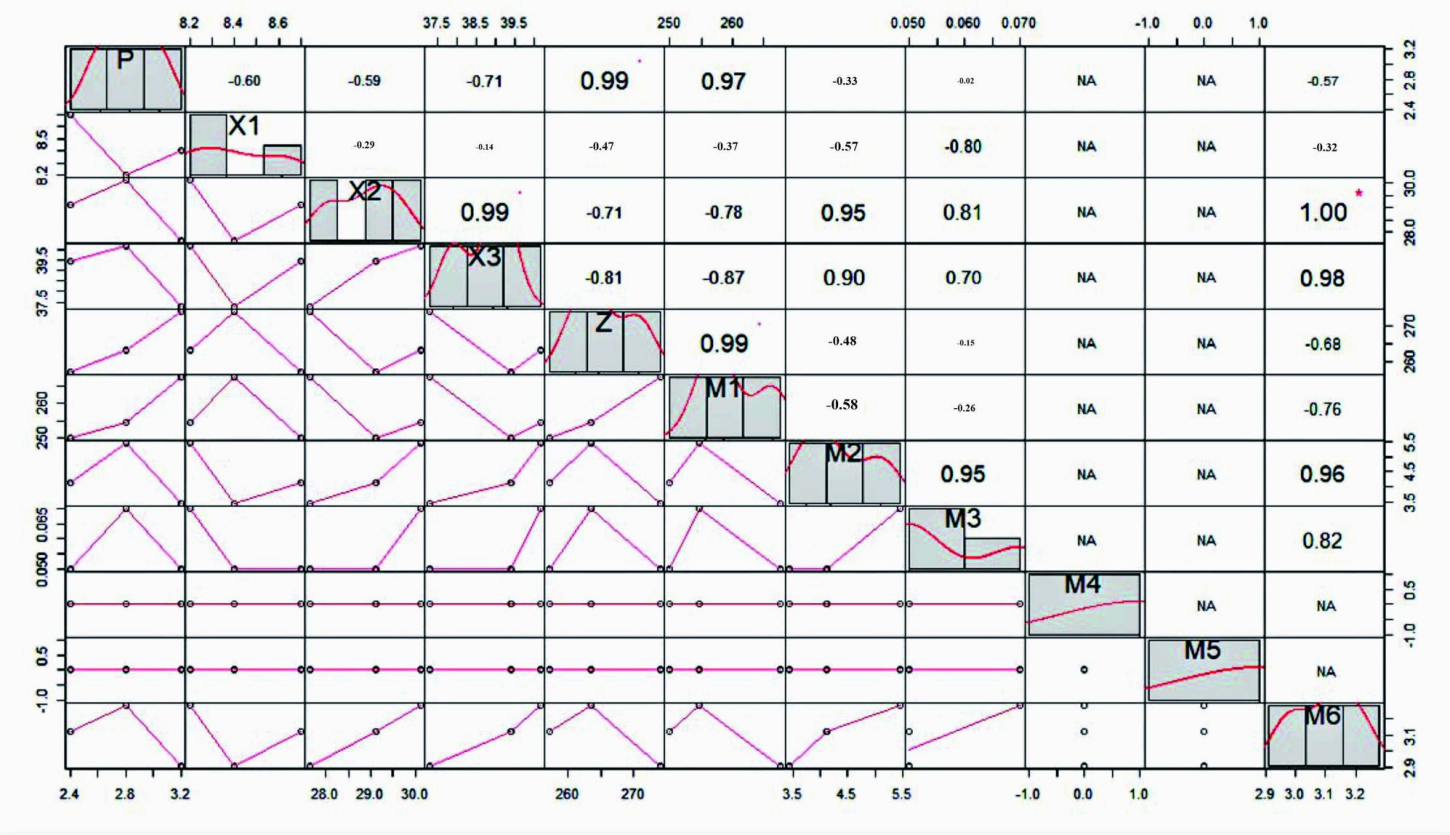
Correlation analysis between root diameter class and soil lead rate and swelling rate under Medicago treatment. P: swelling rate of soil; X1, X2, X3: the line shrinkage of the minimum, maximum and inflection points of suction; M1, M2, M3, M4, M5,M6: respectively denote the root lengths of 0<L< = 0.5mm, 0.5<L< = 1.0mm, 1.0<L< = 1.5mm, 1.5<L< = 2.0mm, 2.0<.L.< = 2.5mm, 2.5<.L.< = 3.0mm; Z:and total root systems; * p < 0.05 and ** p < 0.01.

As can be seen from Fig 8, the swelling rate of soil under Medicago treatment did not correlate with the root length of different diameter classes of Medicago. The line shrinkage rate at the suction value of soil water loss inflection point was significantly correlated with the root length in the range of 2.5<L≤3.0mm diameter class, so the suction was not sufficient to compress the main root system in the diameter class of 2.5<L≤3.0mm when the suction value was small. As the suction value gradually increased, the line shrinkage rate at 0.7 bar suction was significantly correlated with the length of the main root system in the range of 2.5<L≤3.0mm diameter class. There was a significant correlation between soil linear shrinkage and the length of the primary root system.

**Fig 9:**
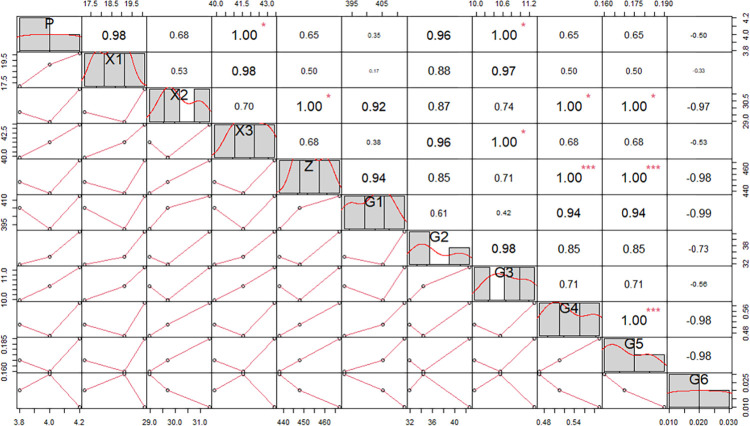
Correlation analysis between root diameter class and soil linear shrinkage and swelling rate under Cynodon dactylon treatment. P: swelling rate of soil; X1, X2, X3: the line shrinkage of the minimum, maximum and inflection points of suction; G1, G2, G3, G4, G5, G6: respectively denote the root lengths of 0<L< = 0.5mm, 0.5<L< = 1.0mm, 1.0<L< = 1.5mm, 1.5<L< = 2.0mm, 2.0<.L.< = 2.5mm, 2.5<.L.< = 3.0mm; Z:and total root systems; * p < 0.05 and ** p < 0.01.

As can be seen from Fig 9, the swelling rate of the soil under the Cynodon dactylon treatment was significantly correlated with the root length within the 1.0<L≤1.5 mm diameter class range of Cynodon dactylon. The root length in the 1.0<L≤1.5mm diameter range accounted for 2.35% of the total root length, which was greater than that in the 1.5<L≤3.0mm diameter range and significantly less than that in the 0<L≤1.0mm diameter range, but the total root length in this diameter range significantly influenced the soil swelling and shrinkage characteristics.The linear shrinkage rate at the inflection point of soil water loss was significantly correlated with root length in the 1.5<L≤2.0mm and 2.0<L≤2.5mm diameter ranges. At a maximum suction value of 10 bar, line shrinkage correlated significantly with root length in the 1.0<L≤1.5 mm diameter range. In summary, as the suction value increases, the range of diameter classes contributing to the effect of soil line shrinkage decreases.

### Effects of different plant roots on soil characteristics

#### Study on changes of soil particle size under different plant roots

**Fig 10:**
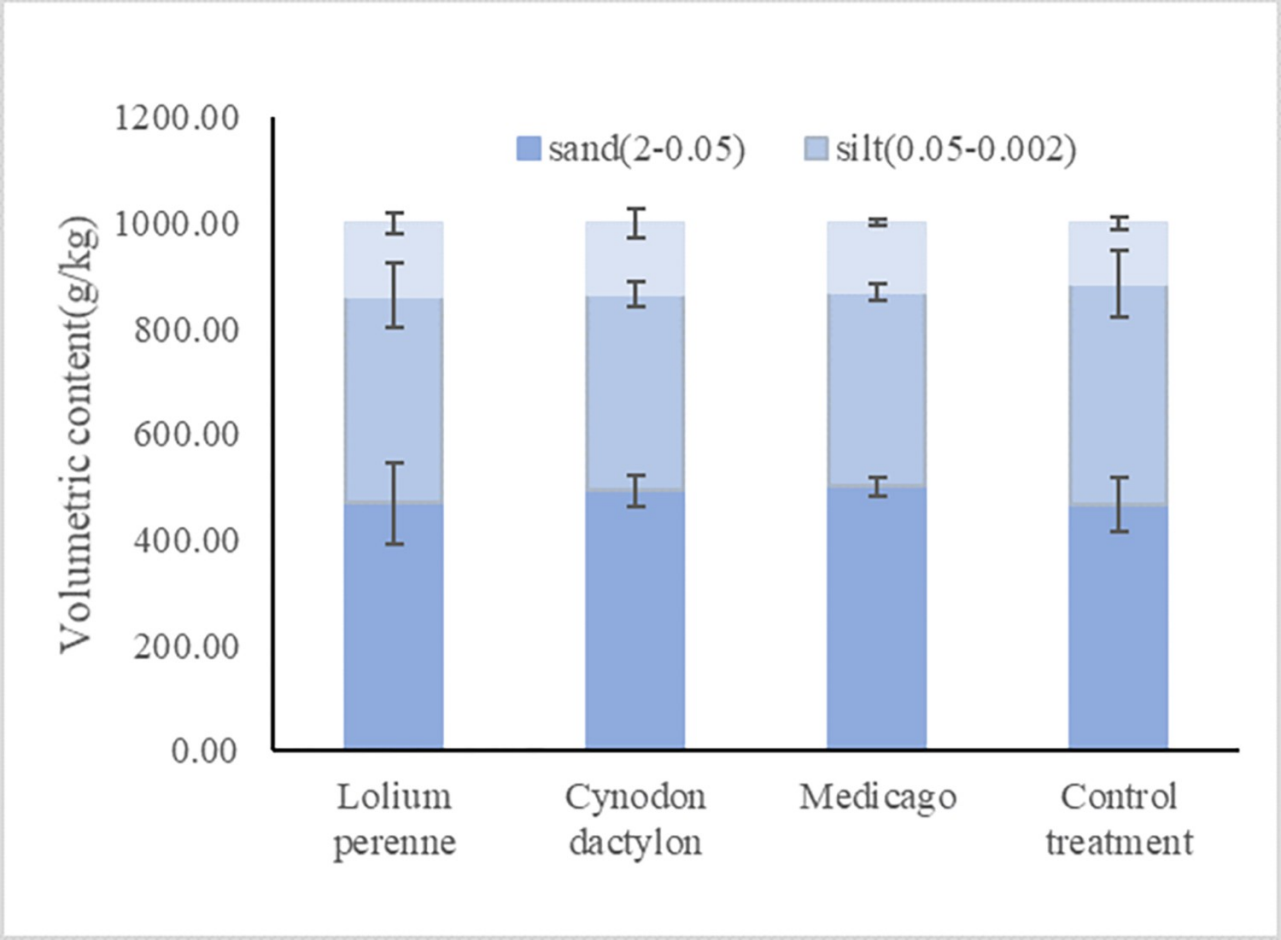
Content of clay, silt and sand particles in the soil under different treatments.

The root extension penetration and root secretion effect, which dispersed and bonded soil particles, influenced the soil structure and soil mechanical composition. The distribution of soil particle size under different treatments is shown in the Fig 10, from which it can be seen that the pattern of influence of different crop growth on soil texture is more obvious. Under the Lolium perenne treatment, the content of powder particles (0.05–0.002) in the soil was reduced by 7% compared with the blank, under the Cynodon dactylon treatment, the content of powder particles (0.05–0.002) in the soil was reduced by 12% compared with the blank, and under the Medicago treatment, the content of powder particles (0.05–0.002) in the soil was reduced by 14% compared with the blank. The content of clay particles (<0.002) in the soil increased by 21% compared to the blank under the Lolium perenne treatment. The content of clay particles (<0.002) in the soil increased by 17% compared to the blank under the Cynodon dactylon treatment, and by 15% compared to the blank under the Medicago treatment.

According to the analysis of soil texture under different treatments, it can be seen that the content of powder particles in the soil were significantly decreased, while the content of clay particles in the soil were significantly increased.

#### Study on the change of soil porosity of slope under different plant treatments

**Fig 11:**
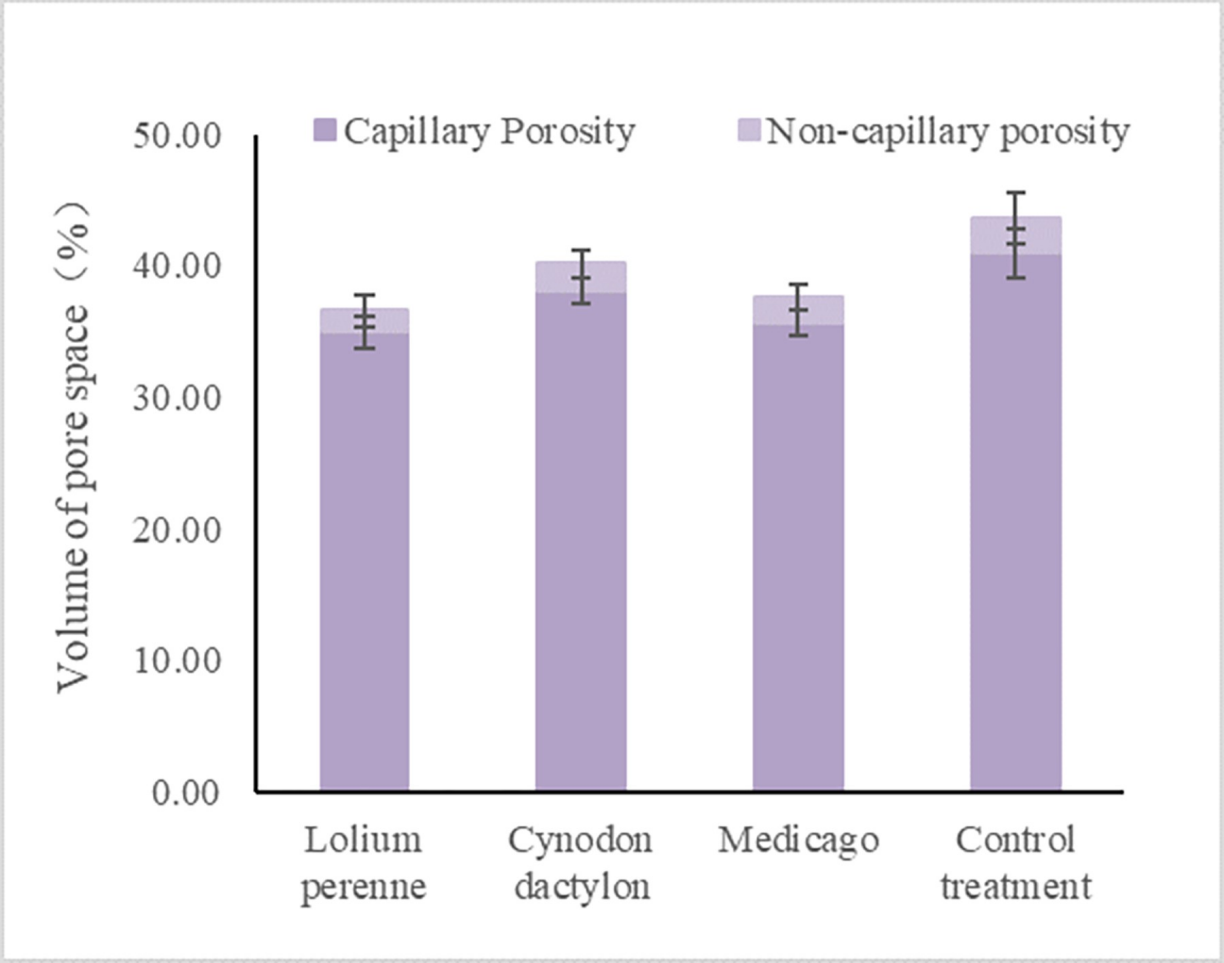
Pore volume in soil under different treatments.

Large differences in soil permeability exist between different plants, which are mainly influenced by the distribution and structural characteristics of the plant root system. Plant roots can effectively improve the soil structure, increase the non-capillary porosity of the soil, reduce the soil bulk, and improve the infiltration performance of the soil. The fine roots are closely distributed in the soil, effectively unclogging the soil pores, improving the soil physicochemical properties and stabilizing the soil structure. As can be seen from the Fig 11, the total capillary porosity of the soil was reduced by 19% for Lolium perenne, 9% for Cynodon dactylon and 16% for Medicago under the three different plant treatments compared with the control treatment. Capillary porosity was reduced by 17% for Lolium perenne, 8% for Cynodon dactylon, and 15% for Medicago compared to the blank. Non-capillary porosity was reduced by 68% for Lolium perenne, 27% for Cynodon dactylon, and 33% for Medicago compared to the control treatment.

According to the analysis, the total porosity, capillary porosity and non-capillary porosity in the soil decreased significantly after planting the crop, in which the decrease of non-capillary porosity under all three plant treatments was significantly higher than that of capillary porosity, and the decrease of the three types of porosity in Lolium perenne and Medicago was greater than that of Cynodon dactylon.

#### Study on the change of soil organic matter of side slopes under different plant treatments

**Fig 12:**
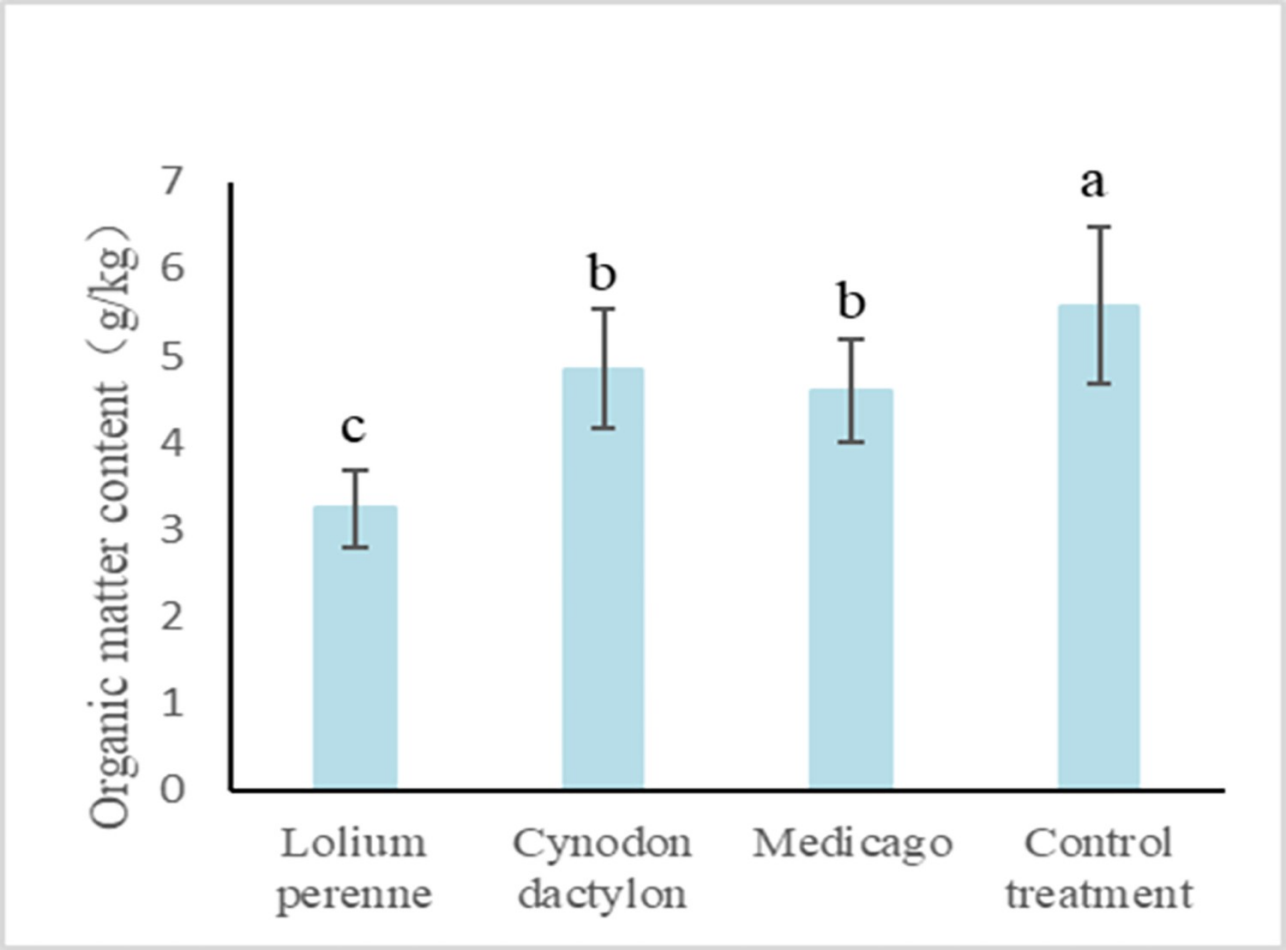
Content of organic matter in soil under different treatments.

Soil organic matter plays a crucial role in maintaining soil structure stability. High organic matter content in soil results in a porous and loose soil structure, which in turn enhances permeability and corrosion resistance while minimizing the adverse effects of soil erosion. It can be seen from Fig 12 that the organic matter content of Lolium perenne, Cynodon dactylon, Medicago growing was reduced by 72%, 15% and 21% compared to the control treatment, respectively. It can be seen that the local original soil has high organic matter content and good quality of soil fertility. After a season of crop cultivation, the organic matter content in the soil under the three crop treatments decreased very significantly without any artificial fertilization measures. Among the three crops, Lolium perenne root system is the most developed and the consumption of organic matter in the soil is the most significant. The following is Medicago, whose main and lateral roots are clear, and the root system is thicker and stronger than the other herbaceous crops, and the consumption of organic matter is greater. Cynodon dactylon is a low-growing plant, with thin and tough culms, the lower creeping ground spread, less developed root system than the previous two, and less consumption of organic matter content in the soil.

#### Micromorphological mechanism of root system effects on soil swelling and shrinking characteristics

**Fig 13:**
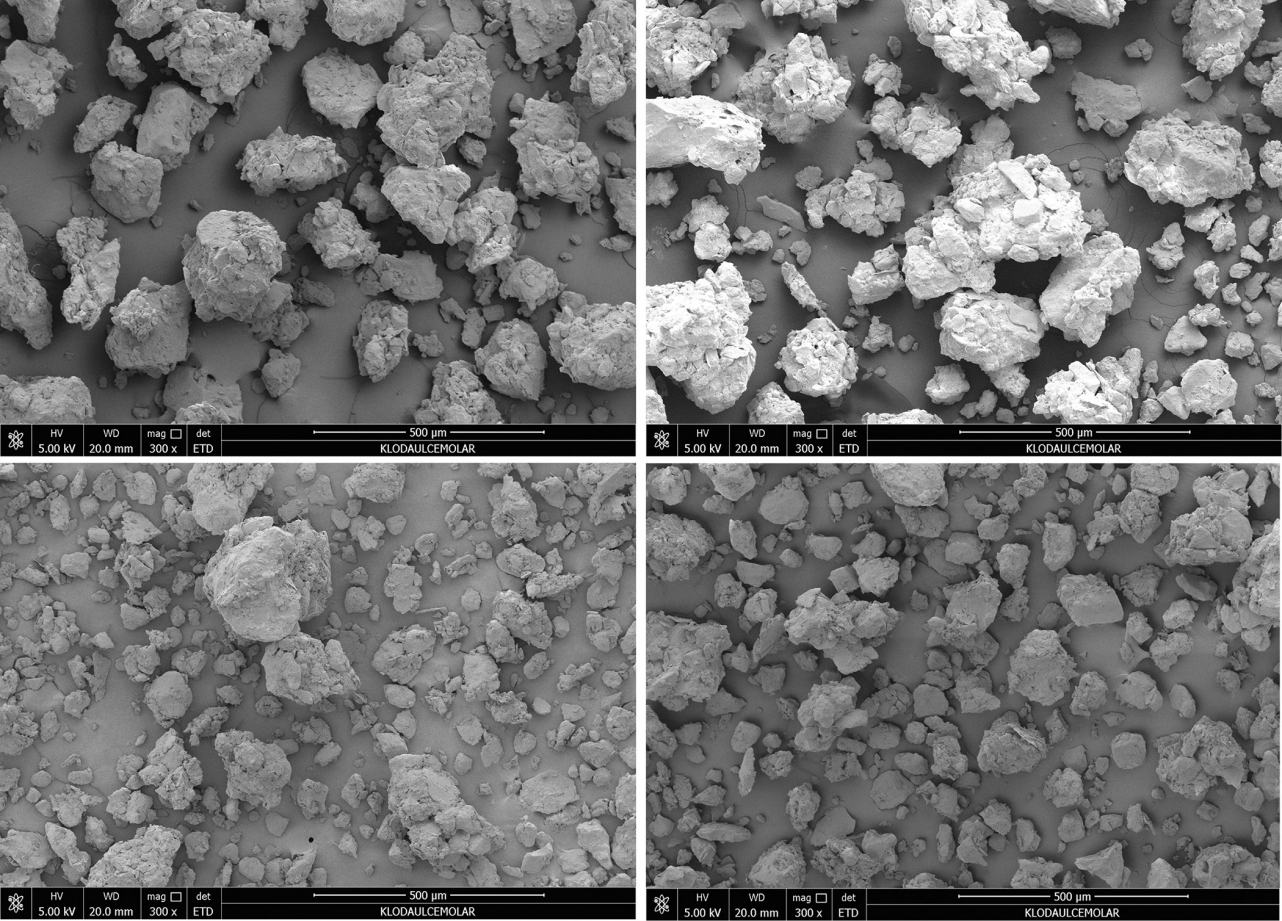
Microscopic morphology of soil under different treatments at 500 μm scale. (a is for Control treatment; b is for Cynodon dactylon; c is for Medicago; d is for Lolium perenne).

**Fig 14:**
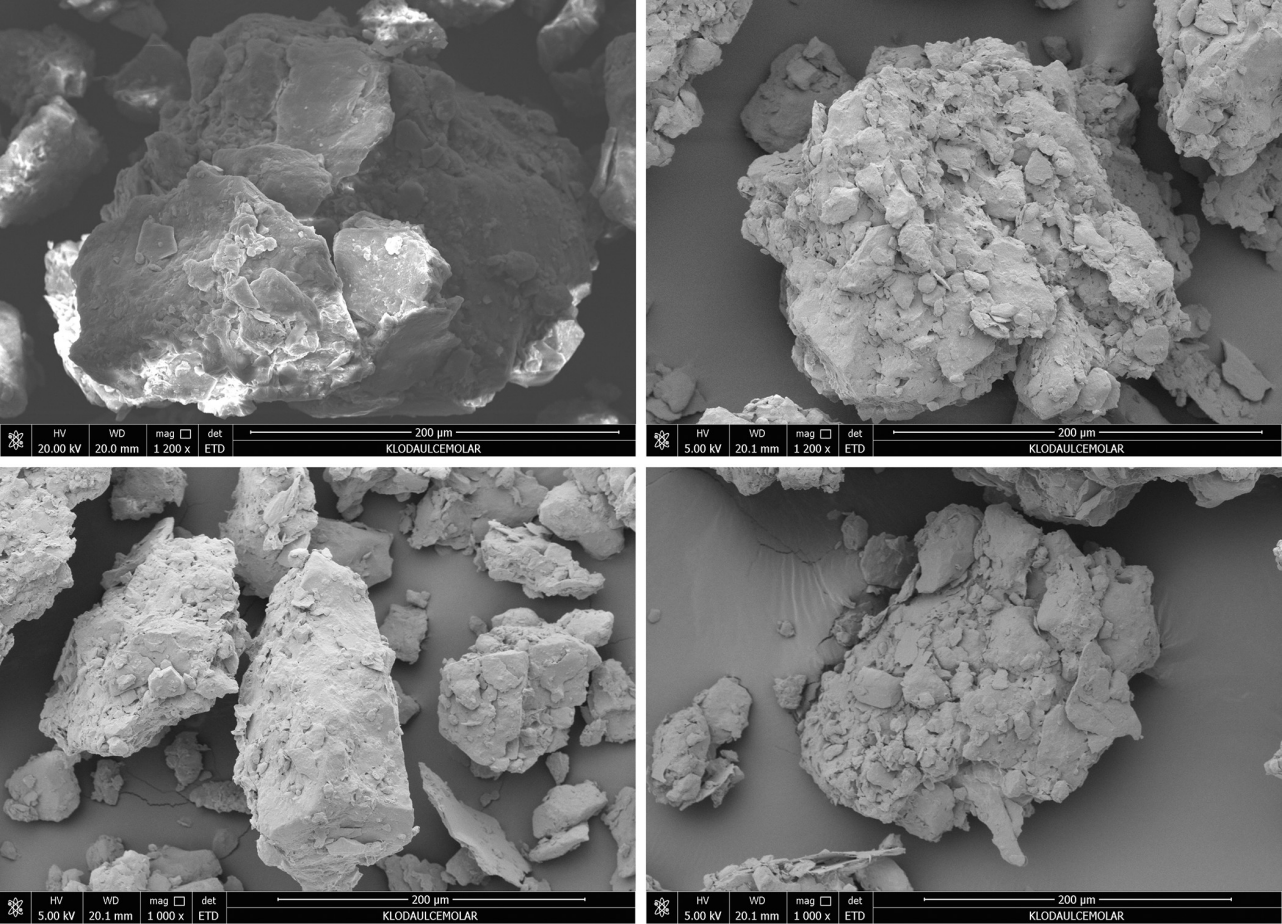
Microscopic morphology of soil under different treatments at 200 μm scale. (a is for Control treatment; b is for Cynodon dactylon; c is for Medicago; d is for Lolium perenne).

In order to analyse the micromorphological characteristics of the root systems of different herbaceous plants in the soil, the morphology of the original soil samples under different treatments was observed by scanning electron microscopy. The images are shown in Figs 13 and 14.

In the analysis of soil micromorphological elements, particles larger than 2μm are skeletal particles, which are mineral in composition and consist mainly of biotite tired rock particles formed during the formation of theparent rock or soil [24, 47, 48]. The skeletal particles are stable and are a component of the soil that does not move or change easily and whose composition is not likely to change in a short period of time under the influence of plant cultivation [20, 49–52]. By observing the micromorphological characteristics of the different treatments at 500 μm, although the composition and properties of the skeletal particles do not change, there are very clear differences in their distribution. As shown in the figure for the Cynodon dactylon, Medicago and Lolium perenne treatments compared to the control, the large agglomerates that were originally more adhesive tended to disperse under the interpenetrating effect of the plant root system. Under the Lolium perenne treatment, the tendency to disperse large agglomerated particles was very significant due to its own redundant root system with long, well-developed roots. In comparison, Cynodon dactylon was less able to locally decompose large soil agglomerated particles due to its shallow root system. Medicago has the weakest local decomposition of large agglomerated particles due to its dominant main root system and the absence of redundant root components.

The fine-grained soil material is mainly made up of soil clay components and humus [53–57]. The fine-grained material of the soil is almost entirely made up of clay minerals, which aggregate to form the soil matrix [58]. The electron microscope images show differences in the micro-basement structure of the soil under the different treatments. Under the control treatment, the surface of the soil particles was relatively smooth, and traces of natural rock weathering were evident in the basal structure. However, there are significant differences between the three plant root treatments compared to the control. The coarse grained debris particles were arranged in a generally parallel pattern to form a mounded basal structure due to the influence of the plant root system [59, 60].Because of the different characteristics of the herbaceous root system, the basal structure was more complex under the Cynodon dactylon and Lolium perenne treatments due to the extension and entanglement of the herbaceous root system, and the surface structure of the soil particles was significantly more concave and convex in a mounded structure, while the basal structure was weaker under the Medicago treatment.

The electron microscope images show that the soil microstructure under the control treatment is gelatinous and compact, with the surface largely free of debris. However, in the soils of Lolium perenne and Cynodon dactylon treated, different types of debris aggregation—embedded debris microstructures were observed on the surface of the soil particles under the plant root system. Under the Medicago treatment, which is characterised by the dominance of the main roots, the surface layer of the soil particles shows a gelatinous compacted—with a small amount of debris accumulation—type of soil microstructure.

In this study, only basal characteristic formations in the soil that are susceptible to the effects of plant growth were analyzed. As shown in the Fig 13, the soil basal characteristic formations were mechanically deformed under all three plant compared to the control treatment.The surface layer of the granules under the Cynodon dactylon and Lolium perenne treatments had distinct basal characteristic formations that differed from the control soil basal formation, with a clear curved moon, filamentous and bow-shaped basal formation in the Fig 12. Under the Medicago treatment, similar basal formation was also present, but the amount and abundance of basal formation was not pronounced. The formation of this type of basal knot formation correlated strongly with the entanglement effect of herbaceous root.

## Discussion

### Root characteristics of different plants

The research displayed distinct morphological characteristics of three herbaceous plants. Lolium perenne exhibited a substantial root system consisting of dense and diverse small, shallow roots. The roots of Lolium perenne were primarily found in the range of 0 < L≤1.0mm and were notably slender. Among these, roots between 0 < L≤0.5mm constituted the majority of the root distribution in Lolium perenne, resulting in the highest root length and root surface area density. Medicago is a plant with a straight taproot measuring between 2.5 < L ≤ 3.0mm. This taproot serves as the main axis for the entire root system in the soil. However, in clay soil with a tendency to swell, the growth of lateral roots is limited. Consequently, Medicago exhibits the lowest values for root length, root surface area, and root weight compared to the other two herbaceous plants. Cynodon dactylon exhibits a robust root spread with well-developed roots, distributed within the range of 0~3.0 mm. Despite having a lower root length and surface area compared to Lolium perenne and Medicago, Cynodon dactylon displays a significantly higher root weight density. The difference in root length index among the three herbaceous plants was found to be highly significant. However, no clear trend was observed in root weight density. This can be attributed to the variation in root morphology among the three plants. Lolium perenne exhibits a rich root system with small root diameter but redundant root system. On the other hand, Medicago has a stout root system with a dominant main root, while Cynodon dactylon has a thicker root system that spreads prostrate, resulting in smaller root length per unit area.

The number of root tips, root forks, and root crosses of the three herbs were ranked as follows: Lolium perenne > Cynodon dactylon > Medicago, with significant differences. Lolium perenne exhibited a wider distribution range in soil and a stronger metabolic capacity in root tips, Which is consistent with the results of previous studies [61]. In the Shangluo area, the soil quality is characterized by stickiness and a high content of soil powder. As the crops grow, the root exudates gradually increase, promoting the formation of colloidal particles and consequently increasing the clay content. The results showed that treatment with Lolium perenne had the best effect on improving soil grain size ratio and significantly improved soil texture, followed by Cynodon dactylon. On the other hand, the main root system of Medicago mainly developed vertically, with low numbers of root tips, root forks and root crosses, resulting in certain limitations on its ability to improve soil texture.

### Effects of different plant roots on soil slope stability

The water holding capacity of different herbs in soil was found to be in descending order: Medicago, Lolium perenne, and Cynodon dactylon.The root shape, distribution, and diameter grades of herbaceous plants vary considerably. As suction values increase, the fine and redundant roots of Lolium perenne become compressed and squeezed. The shrinkage of soil lines is greater under Lolium perenne treatment compared to Medicago treatment. Cynodon dactylon, being a shallow root plant, has surface roots that are connected at obvious points, resulting in reduced binding effect with the surface soil. Consequently, under strong suction, the soil shrinks and the surface roots become exposed. As the suction value continues to increase, the linear shrinkage rate of soil treated by Cynodon dactylon is larger than that of the control treatment. Conversely, linear soil shrinkage was found to be smaller in Medicago and Lolium perenne treatments compared to control treatments at higher suction values.But, there was no significant difference between the two treatments. This indicates that the effect of plant roots on soil contraction is not noticeable under ultimate suction conditions. The application of herbaceous plants in slope protection has a positive effect on the hydraulic holding capacity of slope soil, which can resist the hydraulic erosion caused by natural forces [62, 63]. Therefore, the roots of these plants play a significant role in the application of plant slope protection in soil slope.

The soil under different herb treatment was saturated after 5 hours and no longer absorbed water. The instantaneous expansion rate under control treatment was higher than that under the three plant treatments at all time periods. Compared with the control treatment, soil swelling rate under the three herb treatments decreased by 24~36%. In the application of plant slope protection, herb roots had a significant effect on alleviating soil swelling in the expansive soil area.

### Correlation analysis of different plant roots and soil slope stability

Different herbaceous root morphology had different effects on soil contractility. The impact of herb root morphology on soil shrinkage is contingent on external stress factors. In the presence of natural stress, herb roots exhibit greater resistance to hydraulic erosion of slope soil, resulting in reduced soil shrinkage and improved soil water retention capabilities. At the point where water loss reaches its peak, there was a notable positive correlation between the coarse diameter roots, ranging from 1.5 to 3.0mm, and soil shrinkage. Additionally, at the maximum suction value, there was a significant correlation between the linear shrinkage of soil and the length of roots with a diameter between 1.0 and 1.5mm. The degree of influence of herb root morphology on soil shrinkage depends on external stress. Herb roots can effectively resist hydraulic erosion of slope soil and reduce soil shrinkage, leading to improved soil water retention [13].

The three herbaceous plants showed a reduction in soil swelling rate, with a decrease range of 0.6%-1.8% under different treatments. The soil’s pores, which are typically 0.03 to 0.1mm in size, serve as the primary pathways for air and water movement. The roots of the three plant species examined in this study were found to have a significant presence in the diameter range of 0 < L≤1.5mm. The presence of herbaceous plants in soil can lead to a reduction in water channels in soil macropores, thereby decreasing the water absorption effect of the pores. As a result, the soil expansion rate is reduced under the influence of these plants. However, a notable positive correlation was found between the length of coarse roots in herbaceous plants and the rate of soil expansion. The expansion rate was observed to be relatively high when the root length of plants ranged from 1.0mm to 1.5mm. The analysis of root morphology in three herbs revealed that Medicago, which has taproots, occupied a significant number of soil macropores in the loop cutter. This resulted in a decrease in water absorption performance of soil pores, causing the expansion rate to be the lowest under Medicago treatment. The root system of Lolium perenne is primarily concentrated in soil pores with a diameter of 0 < L ≤ 0.5mm, with the roots closely associated with small macropores in the soil. Additionally, the root length is quite large, occupying a significant portion of the larger soil pores, which can reduce the water absorption performance of these pores. Under Lolium perenne treatment, the expansion rate was significantly decreased compared to the blank treatment, but not to the same extent as the Medicago treatment. Cynodon dactylon has a creeping root system with shallow roots, fewer roots in the cutting ring than Lolium perenne, and a smaller root diameter than the main root diameter of Medicago root system. As a result, it has the highest expansion rate among the three herbaceous plants under this treatment. Research has shown that the shear test of undisturbed soil in different regions presented that the root content are important factors affecting the shear strength of soil [63]. The research also confirms this view. In summary, the impact of herb roots on soil slope expansibility can be attributed to two main factors—root distribution per unit volume and root diameter. These factors play a crucial role in determining the amount of macropores present in the soil, which in turn affects its expansibility and ability to transfer water.

### Effects of different plant roots on soil characteristics

The presence of herbaceous plant roots has been found to have a positive impact on soil texture. In particular, the sand content is reduced while the silt content is significantly decreased and the clay content is increased. Additionally, the planting of herbaceous crops has been shown to increase vegetation coverage on soil slopes, which in turn weakens water and wind erosion and reduces the loss of fine particles. The growth of herbaceous plants, such as Lolium perenne and Cynodon dactylon, with their hairy roots and abundant root systems, has been shown to significantly improve soil structure by promoting the formation of fine particulate matter. This is due to the increased production of root exudates and the gradual increase in colloidal and clay content in the soil. Additionally, the planting of herbaceous plants can disperse large particulate matter in the soil, further contributing to soil improvement. A research has shown that shoot ratios in ryegrass and alfalfa were significantly affected by the soil texture [64]. This research not only confirms the above views, but also reveals the effect of plant roots on soil texture.

The layout of root growth in various plants has a significant impact on the soil porosity of soil slope. The total porosity, capillary porosity, and non-capillary porosity of soil decrease significantly as a result. Among the plants studied, Lolium perenne, a herb with well-developed roots, had the greatest impact on reducing the total porosity, capillary porosity, and non-capillary porosity of soil, followed by Medicago. Cynodon dactylon had the least impact. The study found that the decrease in non-capillary porosity was significantly greater than that of capillary porosity. Lolium perenne, which belongs to the grass family, has a highly developed root system with numerous root hairs that occupy a large number of soil pores. The fine and dense roots intertwine in the soil, helping to consolidate it and prevent it from being easily dispersed by rain or flood. This enhances the soil’s ability to resist erosion. The presence of Medicago’s taproot also had a significant impact on reducing soil porosity around it. In contrast, Cynodon dactylon, with its spread growth, has small root clusters and less root biomass per unit area, resulting in little influence on soil capillary and non-capillary porosity [65]. The impact of herbaceous plant roots on soil porosity varies based on their root characteristics, and the spatial distribution of root growth has a significant effect on soil porosity. Those with a higher density of developed and robust roots per unit area have the greatest effect on soil porosity. Once crops are planted, the roots of these plants help to bind and consolidate loose soil that may have been eroded by wind and water. This makes it more resistant to further erosion caused by rain or floods, ultimately enhancing the stability of soil slopes [66, 67].

The content of soil organic matter was found to be reduced after treatment with three herbaceous plants as compared to the control treatment. This phenomenon is inconsistent with relevant research [68]. Prior to planting, the soil surface had only scattered weeds and the organic matter in the soil was relatively stable. Sampling analysis was conducted at the peak of herb growth after planting. Lolium perenne exhibited the most developed root system and consumed the most organic matter in the soil. Medicago, with its clear taproot and lateral roots, had a stronger root system than other herbaceous crops and consumed a greater amount of organic matter as well. Cynodon dactylon is a plant that grows close to the ground and spreads by creeping. It has a less developed root system compared to the previous two plants mentioned and consumes less organic matter from the soil. To examine the fundamental characteristics of herbal plants, samples were collected during their peak growth period when the roots are most robust. However, since a significant portion of the biomass is extracted as samples, the quantity of plant residues introduced into the cultivated soil is lower compared to natural soil. Consequently, this research investigates the potential adverse impact of crop root growth on soil organic matter. The experiment layout had a significant impact on the surface soil due to the planting measures. The interpenetration of plant roots accelerated the decomposition rate of organic matter, resulting in a faster loss of organic matter within the first year of reclamation. In terms of the economic viability of green slope protection technology, herb planting does not involve the use of auxiliary fertilizers. As a result, during the early stages of planting, the consumption of organic matter in the soil exceeds the accumulation of plant root secretions and residues, leading to an overall decline in organic matter content. With the increase in planting years, there is a gradual development of herb roots and a decline in herb rhizomes, which may result in an increasing trend in the content of organic matter in the soil [68].

### Micromorphological mechanism of effects of different plant roots on soil expansion and shrinkage characteristics

Based on SEM observation, it was found that the interpenetration of plant roots effectively dispersed the large clumps with strong viscosity in all three treatments, as compared to the control treatment. The impact of Lolium perenne on large agglomerated particles is readily apparent. Soils treated with both Cynodon dactylon and Lolium perenne displayed a more intricate basal structure, with soil particle surfaces noticeably raised and mound-like due to the entangling and extension of herbaceous plant roots. The soil treated with Medicago had a weaker structure compared to the soil treated with Lolium perenne and Cynodon dactylon. The treatments of these two plants resulted in distinct debris aggregation and soil microstructures embedded on the surface of soil particles. Due to the dominance of Medicago’s taproots, the soil’s surface layer is characterized by a densely packed microstructure of soil particles, with minimal debris accumulation. The intricate network of roots among herbaceous plants aids in the dispersal of large clumps of soil [69]. The morphology of plant roots plays a significant role in shaping and enriching the soil’s binding structure. A well-developed root system can facilitate the decomposition of fine particulate matter in the soil and enhance the richness of soil micromorphology.

## Conclusions

1. Lolium perenne exhibits a substantial root system consisting of numerous small shallow roots. In contrast, Medicago, being a plant with a straight root structure, possesses distinct advantages in terms of taproot development. Cynodon dactylon, demonstrates robust rhizomes and well-developed roots. Lolium perenne exhibiting significantly higher root length density and root surface area density compared to Medicago and Cynodon. Cynodon dactylon has a higher root weight density than Lolium perenne and Medicago. Lolium perenne had the highest number of root tips, root forks and root crosses, and the metabolic capacity of root tips was stronger.
2. Herbaceous roots have been found to enhance the water holding capacity of soil slopes. The water holding capacity in descending order is Medicago, Lolium perenne, Cynodon dactylon. When subjected to natural stress, herbaceous roots exhibit improved resistance to hydraulic erosion of slope soil, thereby reducing soil shrinkage. The impact of herbaceous plant roots on soil swelling can be attributed to their ability to reduce water channeling in soil macropores. The effectiveness of this reduction is primarily determined by the distribution and diameter of the roots per unit volume. Longer roots with larger diameters are more effective in mitigating soil swelling.
3. The plant roots reduce the loss of fine particles, and the surface of the soil slope that was originally loose due to wind erosion and water erosion becomes compact due to root consolidation, which enhances the slope soil resistance to water erosion and wind erosion. The promotion in root interpenetration resulted in the formation of fine particulate matter, leading to an increase in the colloidal content and clay content in the soil due to the increased root exudates. Due to the implementation of herbaceous planting and management practices in the initial stage, the topsoil of the soil became mixed, resulting in an increase in the mineralization of organic carbon in the soil. As the herbaceous roots gradually developed and the herb rhizomes decayed over time, there may have been an upward trend in the organic carbon content of the soil. In the application of green herbaceous plant slope protection technology, it is advisable to use less disruptive planting methods during the initial planting phase. This means avoiding deep turning, hole sowing, and other planting management methods in order to minimize carbon emissions caused by the initial planting. The entangling and interpenetration of herbaceous plants’ root system can effectively disperse large sticky clumps. Furthermore, a well-developed root system contributes to the decomposition of soil fine particulate matter and enhances the richness of soil micromorphology.
4. Herbaceous plants play a crucial role in soil consolidation and slope protection. Lolium perenne and Medicago exhibit a favorable slope protection effect during the initial stages of growth, attributed to their distinctive root characteristics. However, Cynodon dactylon, with its creeping roots, has a relatively smaller root volume and diameter per unit volume within a short period of time, resulting in a slightly weaker protection effect on soil slopes. Plant roots collectively enhance the resistance of soil slope to water and wind erosion through the interweaving and consolidation of roots, dispersing large clay particles, altering soil porosity, enhancing soil water retention capacity, and reducing soil water infiltration, thereby achieving slope protection. The research provides support and guidance for understanding the mechanisms through which roots influence slope stability and for the development of plant-based slope protection technologies.

## Supporting information

S1 Data (XLSX)
